# A *CTNNA3* compound heterozygous deletion implicates a role for αT-catenin in susceptibility to autism spectrum disorder

**DOI:** 10.1186/1866-1955-6-17

**Published:** 2014-07-10

**Authors:** Elena Bacchelli, Fabiola Ceroni, Dalila Pinto, Silvia Lomartire, Maila Giannandrea, Patrizia D'Adamo, Elena Bonora, Piero Parchi, Raffaella Tancredi, Agatino Battaglia, Elena Maestrini

**Affiliations:** 1 Department of Pharmacy and Biotechnology, University of Bologna, via Selmi 3, Bologna 40126, Italy; 2 Seaver Autism Center for Research and Treatment, Icahn School of Medicine at Mount Sinai, New York, NY 10029, USA; 3 Department of Psychiatry, Icahn School of Medicine at Mount Sinai, New York, NY 10029, USA; 4 Department of Genetics and Genomic Sciences, Icahn School of Medicine at Mount Sinai, New York, NY 10029, USA; 5 The Mindich Child Health and Development Institute, Icahn School of Medicine at Mount Sinai, New York, NY 10029, USA; 6 Dulbecco Telethon Institute at San Raffaele Scientific Institute, Division of Neuroscience, Milan 20132, Italy; 7 Vita-Salute San Raffaele University, Milan 20132, Italy; 8 Unit of Medical Genetics, Department of Medical and Surgical Sciences, S. Orsola-Malpighi Hospital, University of Bologna, Bologna 40138, Italy; 9 IRCCS Institute of Neurological Sciences, Bologna 40139, Italy; 10 Department of Biomedical and Neuromotor Sciences, University of Bologna, Bologna 40139, Italy; 11 Stella Maris Clinical Research Institute for Child and Adolescent Neuropsychiatry, Calambrone, Pisa 56128, Italy

**Keywords:** Autism spectrum disorder (ASD), *CTNNA3*, αT-catenin, Alpha T-catenin, Cell adhesion, DNA copy number variants

## Abstract

**Background:**

Autism spectrum disorder (ASD) is a highly heritable, neurodevelopmental condition showing extreme genetic heterogeneity. While it is well established that rare genetic variation, both *de novo* and inherited, plays an important role in ASD risk, recent studies also support a rare recessive contribution.

**Methods:**

We identified a compound heterozygous deletion intersecting the *CTNNA3* gene, encoding αT-catenin, in a proband with ASD and moderate intellectual disability. The deletion breakpoints were mapped at base-pair resolution, and segregation analysis was performed. We compared the frequency of *CTNNA3* exonic deletions in 2,147 ASD cases from the Autism Genome Project (AGP) study versus the frequency in 6,639 controls. Western blot analysis was performed to get a quantitative characterisation of Ctnna3 expression during early brain development in mouse.

**Results:**

The *CTNNA3* compound heterozygous deletion includes a coding exon, leading to a putative frameshift and premature stop codon. Segregation analysis in the family showed that the unaffected sister is heterozygote for the deletion, having only inherited the paternal deletion. While the frequency of *CTNNA3* exonic deletions is not significantly different between ASD cases and controls, no homozygous or compound heterozygous exonic deletions were found in a sample of over 6,000 controls. Expression analysis of Ctnna3 in the mouse cortex and hippocampus (P0-P90) provided support for its role in the early stage of brain development.

**Conclusion:**

The finding of a rare compound heterozygous *CTNNA3* exonic deletion segregating with ASD, the absence of *CTNNA3* homozygous exonic deletions in controls and the high expression of Ctnna3 in both brain areas analysed implicate *CTNNA3* in ASD susceptibility.

## Background

Autism spectrum disorder (ASD) is a group of related lifelong neurodevelopmental conditions that affect about 1 in 110 individuals [1]. ASD is characterised by defects in communication, impaired reciprocal social interaction, repetitive behaviours and restricted interests, with onset before age 3 years. A peculiarity of ASD is a gender bias, with males having a greater than threefold higher risk for ASD as compared to females [2].

Despite the high heritability of ASD (approximately 90%), its basis remains poorly explained by common genetic risk variants [3]. Genome-wide association studies (GWAS) have so far provided only tenuous evidence for individual common variants that affect risk of ASD [3-6], drawing attention to the contribution of rare variants to ASD pathophysiology. Indeed, recent studies have shown that rare genomic variation, both copy number variants (CNVs) and point mutations, may account for a significant proportion of cases of idiopathic autism [7]. Although CNV and exome sequencing studies suggest that some 10% of ASD subjects carry a *de novo* risk variant, demonstrating that *de novo* genetic variation has an important role in risk for an ASD phenotype, this mechanism is inconsistent with the widely recognized high heritability. Convincing statistical evidence for the role of rare recessive inherited variants in ASD risk comes from two recent studies that applied whole-exome sequencing to a cohort of consanguineous and/or multiplex families and to ASD cases using a population-based approach [8,9]. The contribution of recessive mutations to ASD heritability is supported by the success of homozygosity mapping to identify autism genes in consanguineous families [10], the use of homozygosity mapping as a powerful strategy for filtering whole-exome sequence data [11] and the identification that ASD probands display a much higher degree of haplotype sharing within overlapping homozygous regions compared to parental controls [12].

In the second stage of the Autism Genome Project (AGP) genome-wide study, amounting to approximately 1,600 ASD families [13], we detected one rare compound heterozygous deletion involving the *CTNNA3* gene, encoding αT-catenin, in a proband with autism. CTNNA3 is a member of the α-catenin family and has a crucial role in cell adhesion, one of the major pathways implicated in ASD [4,14-17]. *CTNNA3* has been implicated as a possible autism candidate gene ( https://gene.sfari.org/GeneDetail/CTNNA3#HG). Previous studies reported common single nucleotide polymorphism (SNP) association [4,5] and the occurrence of rare CNVs intersecting *CTNNA3* in ASD cases [18-21]. Therefore, the recessive inheritance pattern seen in this family leads us to hypothesize that total loss of *CTNNA3* may cause the ASD phenotype.

In this study, we genetically characterised the rare *CTNNA3* compound heterozygous microdeletion, performed segregation analysis and further clinical evaluation in this nuclear family, assessed the rate of *CTNNA3* deletions in ASD cases and controls and assessed the protein expression pattern of CTNNA3 in the developing mouse brain. This is the first report of a compound heterozygous exonic deletion in *CTNNA3.*

## Methods

### Characterisation and segregation analysis of *CTNNA3* deletions

The maternal and the paternal exon 11 *CTNNA3* microdeletions segregating in family 3456 were amplified via several polymerase chain reaction (PCR) assays using the Gold Taq polymerase (Life Technologies, Carlsbad, CA, USA). Primer pairs were designed following visual inspection of the Illumina 1 M-duo SNP array data from the second stage of the AGP genome-wide CNV study [13], within GenomeStudio (Illumina, San Diego, CA, USA). The paternal 301-bp and the maternal 949-bp deletion-spanning amplicons were purified using Exosap (GE Healthcare, Little Chalfont, UK) and then sequenced using BigDye Terminator kit v1.1 (Life Technologies) to determine the exact boundaries of the deletions. An additional primer pair that amplifies exon 11 of *CTNNA3* (not-deleted allele) was subsequently used to confirm that the identified deletions in family 3456 were in the heterozygous or homozygous status.

The experimental validation of *CTNNA3* exonic deletions in four other ASD families was carried out by real-time quantitative PCR (qPCR) using Fast SYBR Green (Life Technologies). Each assay was conducted in triplicate, with at least three sets of primers corresponding to the region of interest and another mapping to a control region on *FOXP2* gene at 7q31.1. The number of copies of each amplified fragment was calculated using the ΔΔCt method [22]. The parents and additional affected or unaffected siblings were also tested for inheritance and segregation of CNVs, respectively.

All primer sequences and conditions used for amplification, Sanger sequencing and qPCR are available on request.

Numbering for *CTNNA3* exons is based on the Reference Sequence (RefSeq) NM_013266.

### Population analysis of *CTNNA3* exonic deletion

The ASD samples used here were collected as part of the Autism Genome Project. All diagnostic, clinical and cognitive assessments of these samples were previously described [13,23].

*CTNNA3* exonic deletions in ASD samples were identified as a part of the AGP study [13,23]. Briefly, all *CTNNA3* exonic deletions reported in ASD samples are high-confidence CNVs predicted by intersecting CNV calls from at least two algorithms between iPattern, PennCNV and QuantiSNP. This strategy ensures maximum specificity because each of these algorithms employs unique strategies for CNV calling, allowing their strengths to be leveraged. Previous analysis showed that validation rates were approximately 95% for CNVs identified using this method [23].

Control cohort microarray data include 1,287 unrelated European control subjects from the Study of Addiction: Genetics and Environment cohort (SAGE) [24] genotyped with Illumina Human 1 M-single BeadChip arrays, 1,123 Northern Europeans from the German PopGen project (POPGEN) [25] genotyped on the Affymetrix 6.0 SNP array (Affymetrix, Santa Clara, CA, USA), 1,234 individuals of European decent from the Ottawa River Valley (OHI) [26] genotyped on the Affymetrix 6.0 SNP array, 1,320 European control subjects routinely seen at primary care and well-child clinic practices within the Children's Hospital of Philadelphia (CHOP) Health Care [27] genotyped with Illumina 550 K BeadChip, 435 unrelated European control subjects from the Ontario Colorectal Cancer Case-Control study (OC) [28] genotyped with the Illumina 1 M single array and 1,240 European controls from the NHGR-CIDR Visceral Adiposity Study [29] genotyped on Illumina 1 M-duo BeadChip arrays. For all these control samples (except for the CHOP samples, for which the CNV data are available at http://cnv.chop.edu), the heterozygous state of exonic *CTNNA3* deletions has been determined by inspecting the genotypes and/or plotting B allele freq and log *R* ratios for each region.

Statistical comparison of *CTNNA3* exonic deletion frequencies between ASD cases and controls was performed using Fisher's exact test.

All data from either patients or their caretakers and controls, including the informed consent, were handled in accordance with the local ethical committee's approved protocols and in compliance with the Helsinki declaration.

### *CTNNA3* and *LRRTM3* exon sequencing

All coding exons, intron-exon boundaries and the 5′-UTR of *CTNNA3* and the nested gene *LRRTM3* have been amplified by PCR in all members of four families carrying *CTNNA3* exonic deletions. Primer sequences and PCR conditions used for amplification (20 amplicons for *CTNNA3* and 5 amplicons for *LRRTM3*) are available on request. PCR products were purified using Exosap (GE Healthcare) and then sequenced using BigDye v1.1 (Life Technologies).

### Human brain samples

Human brain samples from the frontal cerebral cortex and cerebellum were obtained from deep frozen (-80°C) slices of two adult control subjects. Written informed consent for research use, given by the patients during life or by their next of kin after death, was available for all human brain tissues used for RNA analyses.

### RT-PCR

Total RNA from human frozen brain tissues (30–40 mg) was extracted using the Qiagen Total RNA kit (Qiagen, Venlo, the Netherlands), and reverse transcriptase PCR (RT-PCR) was performed using the Superscript III First Strand Synthesis SuperMix (Life Technologies) according to the manufacturer's protocol. Two microlitres of complementary DNA (cDNA) was used for testing *CTNNA3* expression in the human frontal cerebral cortex and cerebellum using a forward primer designed in exon 10 (CCAATCATTTGGAAACCTTGTG) and a reverse primer mapping in exon 15 (CTCAATCTCAGCATCCAGCTTA), in order to amplify a cDNA fragment including the coding SNP rs4548513 (pSer596Asn). PCR products were purified and sequenced as described before using primers GTTACGAGCCAGGGGCTTAC and CAAGGTCAGAAACATCCTCCA.

### Western blot analysis

The hippocampus and cortex were dissected from three animals (C57Bl/6 N) at each time point pooled together. Samples were lysed with lysis buffer containing 1% SDS and boiled. Forty micrograms of total proteins were loaded onto a 4%–12% polyacrylamide gel (Life Technologies) and then transferred to a nitrocellulose membrane (Whatman). Filters were hybridized with antibodies against N-catenin (Santa Cruz Biotechnology, Inc., Dallas, TX, USA) or T-catenin [30] or GAPDH (Millipore, Billerica, MA, USA) as housekeeping control and then revealed by using HRP-conjugated specific secondary antibodies (BioRad, Hercules, CA, USA) and ECL (GE Healthcare). ImageJ was used to quantify bands.

Experiments were done according to the animal protocols approved by the Institutional Animal Care and Use Committee San Raffaele (IACUC) (San Raffaele, Milan, Italy) and were approved by the National Ministry of Health, IACUC ID 470. All experiments were carried out in accordance with the guidelines established by the European Community Council Directive of 24 November 1986 on the use of animals in research (86/609/EEC). All efforts were made to minimize animal suffering and to use only the number of animals necessary to produce reliable results.

## Results

### Clinical assessment of family 3456

The propositus was the first child of healthy non-consanguineous parents, born at 39 weeks gestation by spontaneous delivery. During pregnancy, there was no exposure or history of chronic illnesses, alcohol, tobacco or street drugs. Family history was non-contributory. Birth weight was 3,500 g (50th centile), length 52 cm (90th centile) and occipitofrontal circumference (OFC) 34.5 cm (50th centile). Apgar was 8–9. He was breast fed with good suction. He sat unsupported at 9 months and walked alone at 19 months. He babbled at 12 months and was able to pronounce his first words at 30 months. Echolalia was noted. Sleep-wake rhythm was normal. Feeding was selective for semi-solid foods. He was withdrawn from early on. He showed no interest toward his peers and was very passive, with no eye-to-eye contact. His behaviour was characterised by motor instability, low level of frustration tolerance, very poor and repetitive interests, bruxism and, on occasion, motor stereotypies. Metabolic work-up, brain MRI, BAEP and EEG were reportedly normal.

We first saw him at age 4 years 8 months. On examination, there were bilateral epicanthal folds, large and anteverted ears, high arched palate and bilateral pes planus pronatus. Height was 113.5 cm (90th centile), weight 17.5 kg (50th centile) and OFC 50 cm (2nd to 50th centile). Neurological examination showed mild joint laxity. Language was limited to simple sentences, with echolalia. Gesture repertoire was poor. Comprehension was contextual.

When last seen, aged 5 years 6 months, height was 118 cm (90th centile), weight 22 kg (75th to 90th centile) and OFC 50.5 cm (2nd to 50th centile). He had a borderline cognitive impairment (Griffiths Mental Developmental Scales and Leiter International Performance Scale), with performance better than verbal competences. At the ADOS-G and ADI-R, he met criteria for autism on both instruments. At age 5 years, he was started on risperidone. Molecular analysis at the FRAXA/E loci was normal.

His younger sister has normal cognition (WISC-R: TIQ 86; VIQ 90; PIQ 85) with normal social and communication skills. Both parents, heterozygotes for the deletion, do not show any evident cognitive or behavioural impairment.

### Fine-mapping and segregation analysis of *CTNNA3* deletion in family 3456

**Figure 1:**
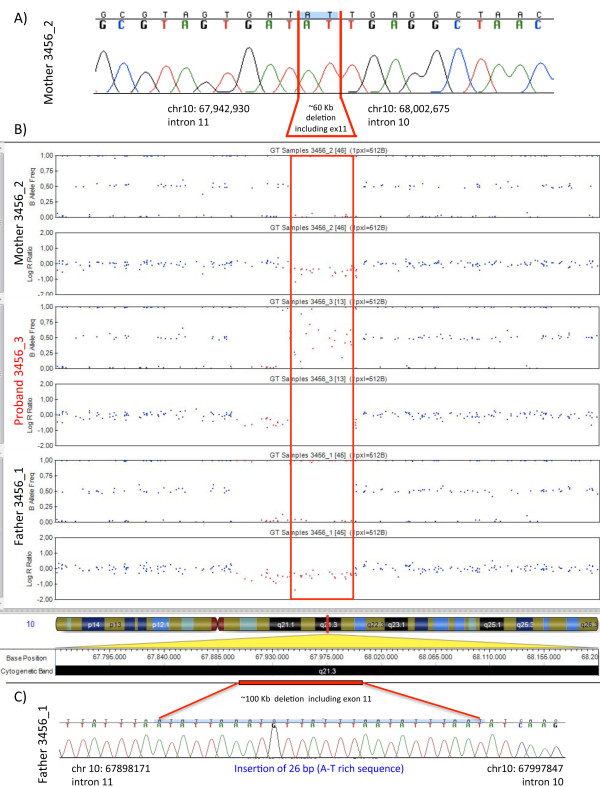
**Detection and breakpoint definition of *CTNNA3 *deletions in the discovery pedigree. (A, C)** Sequence electropherograms with breakpoint-spanning sequences in the mother and in the father, respectively (NCBI build 36 coordinates). In **(A)** the two base pairs (AT), common to both ends, are highlighted in *blue*. **(B)** GenomeStudio plots of the log *R* ratio and B allele frequency data from the 1 M-Duo SNP array for family 3456. The deletions result in a decrease in the log *R* ratio and a lack of heterozygous variants (the expected allelic ratio for heterozygous variants is 0.5). The deleted SNPs are depicted in *red*, and the *red rectangle* indicates the region deleted on both alleles in the proband.

During the second stage of a large genome-wide scan for CNVs carried out by the AGP, we detected an exonic deletion in the *CTNNA3* gene inherited from both parents, each heterozygote for a deletion of slightly different length. After manual inspection of the log *R* ratios and B allele frequencies in GenomeStudio, the minimal deleted region was determined to be from rs12254628 to rs7919336 in the mother 3456_2 and from rs4587626 to rs7077638 in the father 3456_1 (Figure 1B). Using several PCR assays with primers designed to flank the predicted breakpoints, we defined and sequenced the breakpoints of the two slightly different microdeletions carried by the parents of family 3456: the maternal microdeletion encompassed chr10: 67,942,931–68,002,674, while the paternal one spanned chr10: 67,898,172–67,997,846 (National Center for Biotechnology Information build 36 coordinates) (Figure 1A and C).

Since the maternal and paternal deletions have different breakpoints, they cannot be due to a single ancestral event. Sequencing of the deletion breakpoints showed the presence of 2–5-bp microhomology at the junctions of the maternal and paternal deletions, respectively (Additional file 1: Figure S1) as well as the insertion of a 26-bp A-T rich sequence at the paternal deletion junction. These observations, together with the non-recurrent nature of the deletions and the absence of flanking low copy repeats (LCR), suggest that these deletions are likely to be generated through a microhomology-mediated repair mechanism [31].

**Figure 2:**
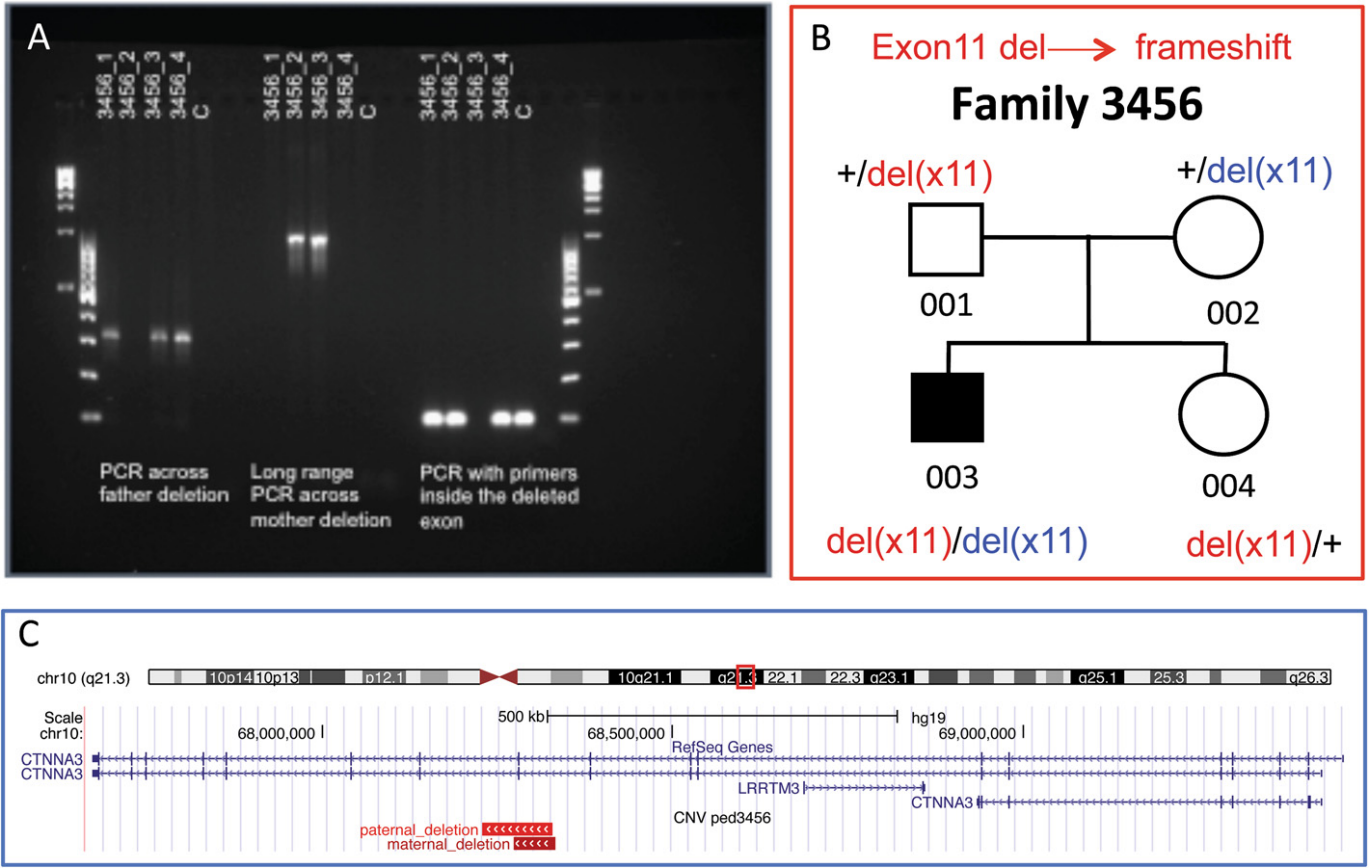
**Further characterisation of *CTNNA3 *deletions in the pedigree 3456. (A)** From the left, results of PCR across father's and mother's deletion breakpoints, respectively: only the allele with the deletion can be amplified and visualized as a band of 301 bp in the father, in the proband and the unaffected sister, and as a band of 949 bp in the mother and in the proband. On the right, the amplification with primers mapping in the deleted exon of *CTNNA3* indicates the presence of at least one allele without the deletion. At both extremities of the gel, 1-kb Plus and 100-bp DNA ladder were loaded. **(B)** The segregation pattern for these two deletions involving *CTNNA3* in the discovery pedigree. Autism is indicated in *black filling*. **(C)** Schematic from the UCSC genome browser. The figure shows the position of the two *CTNNA3* deletions in the pedigree 3456. The region shown corresponds to approximately 1.8 Mb on 10q21.3 (NCBI build 37 coordinates).

The same PCR assays were also used to analyse the segregation of the two microdeletions in the family: while the ASD proband inherited both microdeletions, the unaffected sister inherited only the paternal microdeletion (Figure 2A,B). Both deletions remove exon 11 of *CTNNA3* (Figure 2C): exon 11 is present in both the full-length *CTNNA3* isoforms a (NM_013266 and NM_001127384), which are two transcript variants that differ only for the first 5′ non-coding exon. *CTNNA3* transcripts missing exon 11 are predicted to result in a frameshift, with the introduction of 12 novel amino acids followed by a premature termination codon but also probably inducing nonsense-mediated decay. However, *CTNNA3* was undetectable in blood RNA by RT-PCR, so it was not possible to confirm the functional effect of this exonic deletion.

### *CTNNA3* exonic deletion frequency in ASD cases and controls

Given the discovery of a compound heterozygous deletion of exon 11 in *CTNNA3* in a male proband with autism, we examined the frequency of *CTNNA3* exonic deletions in autism and control populations. To address this issue, we used the existing CNV data on a total of 2,147 European ASD families from the recent AGP genome-wide study (combined sample of stage 1 and stage 2 families) [13] and CNV data on 6,639 European controls [24-29].

We identified a total of 14 additional heterozygous exonic deletions in ASD probands (allelic frequency = 0.37%), thus showing a modest deviation from the Hardy-Weinberg equilibrium (exact test *P* = 0.028). In the control population, we found 43 *CTNNA3* exonic deletions (allelic frequency 0.32%), indicating a comparable frequency between ASD cases and controls (Table 1, *P* = 0.62). Parental information was available for 13 out of 15 ASD families, and in none of these cases, *CTNNA3* exonic microdeletions were *de novo*. The observed exonic deletions are different in position, size, sequence junctions and genomic content, removing one or two exons, spanning from exon 6 to exon 13 of *CTNNA3* (Additional file 2: Table S1). This is in accordance with *CTNNA3* being located in a common fragile site [32], a region characterised by increased genomic instability [33].

**Table 1:** **Frequency of *CTNNA3 *** **exonic deletions in ASD cases and controls**

	**Number of subjects**	**Exonic deletions**	**Exonic deletion frequency (%)**	***P *** **value**^**a**^	**Frameshift deletions**	**Frameshift deletion frequency (%)**	***P *** **value**^**a**^
ASD cases	2,147	16^b^	0.37	0.62	6^b^	0.14	0.56
Controls	6,639	43	0.32		14	0.11	

^a^Fisher's exact test; ^b^Including the two exon 11 deletions in family 3456.

Since deletions that result in a frameshift cause unambiguous loss-of-function alleles, we focused our attention to this class of deletions. Among the observed exonic *CTNNA3* deletions in case and controls (Additional file 2: Table S1), only deletions that remove exon 7, exons 10–11, exon 11 and exon 13 (NM_013266) are predicted to induce frameshifts, but their frequency is not significantly different between cases and controls (Table 1, *P* = 0.56).

### Segregation analysis of *CTNNA3* exonic deletions and mutation screening of *CTNNA3* and *LRRTM3* in four ASD families with affected and/or unaffected siblings

**Figure 3:**
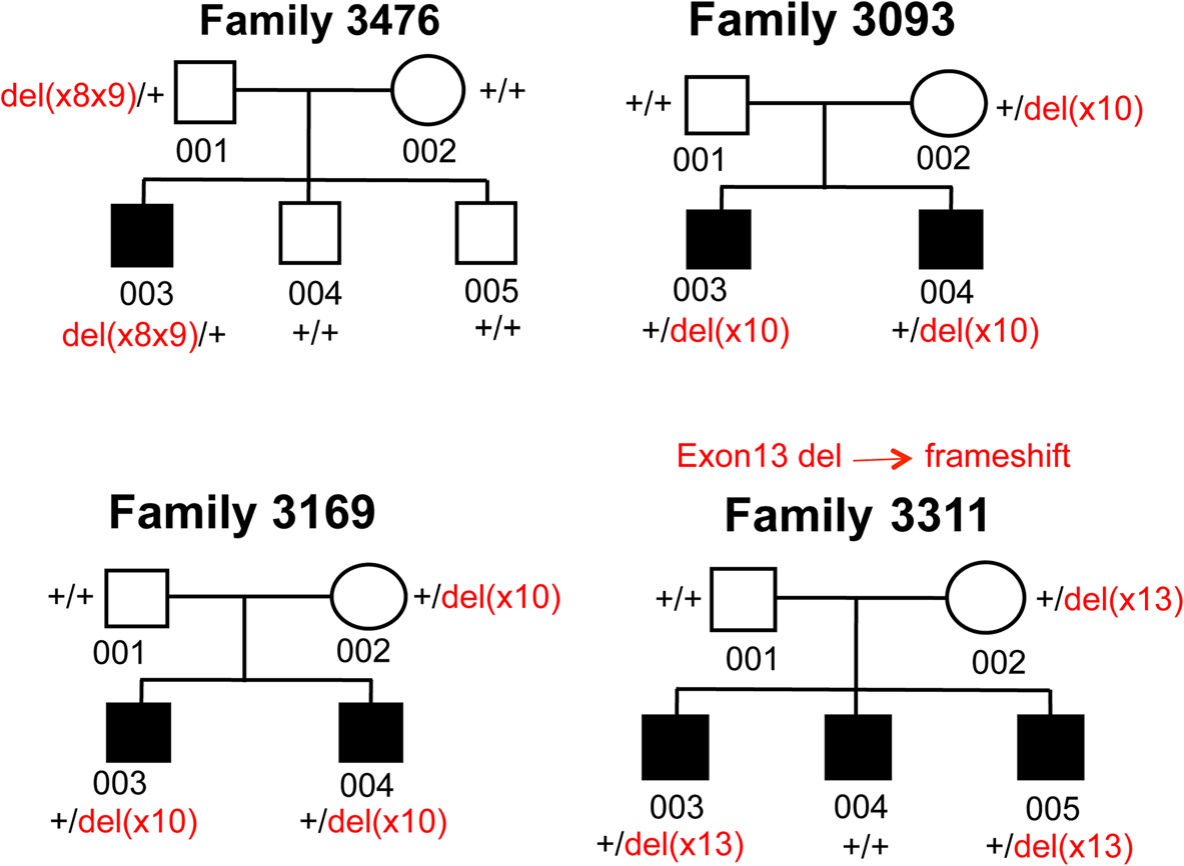
**Pedigree of four ASD families carrying *CTNNA3 *exonic deletions.** *Black filling* indicates ASD diagnosis.

To investigate if *CTNNA3* exonic deletions segregate with the ASD phenotype, we screened the affected and unaffected siblings in three multiplex families and one singleton family for the presence of the identified exonic deletions by qPCR. All *CTNNA3* deletions segregate with ASD phenotype within the family, except for the exon 13 deletion in family 3311 that is transmitted from the mother to only two out of three affected children (Figure 3).

In the hypothesis that the *CTNNA3* deletions could act by unmasking rare variants in the non-deleted allele, we sequenced the entire coding sequence of *CTNNA3* and of the nested gene *LRRTM3* (leucine-rich repeat transmembrane neuronal 3) in all family members of the same four ASD families carrying heterozygous exonic deletions. Sequence analysis did not detect any novel exonic variants in *CTNNA3*, while a previously undescribed missense change (pArg275Ser) was identified in *LRRTM3* in family 3476; however, this missense variant was transmitted from the unaffected father, who also carries the *CTNNA3* deletion, to the affected child. This result is thus not compatible with a two-hit model, since both the *CTNNA3* deletion and the *LRRTM3* missense variant are present in the unaffected father.

### *CTNNA3* expression analysis

We were unable to obtain a reliable amplification of *CTNNA3* and *LRRTM3* transcripts using RT-PCR in blood RNA or Epstein Barr virus (EBV)-transformed cell lines, in accordance with their previously described restricted expression pattern [30]. Therefore, it was not possible to test the functional effect of the heterozygous and homozygous loss of exon 11 in family 3456.

It has been previously reported that *CTNNA3* is subject to genomic imprinting, with preferential monoallelic expression of the maternal allele in placental tissue [34]. Here we investigated the allelic expression of rs4548513, a *CTNNA3* exon 13 coding SNP (pSer596Asn), in the cerebellum and cerebral cortex of two informative heterozygous adult controls. RT-PCR analysis showed high expression levels in the cerebellum and cerebral cortex, and Sanger sequencing of the PCR products showed biallelic expression of *CTNNA3* in both brain areas (Additional file 3: Figure S2).

**Figure 4:**
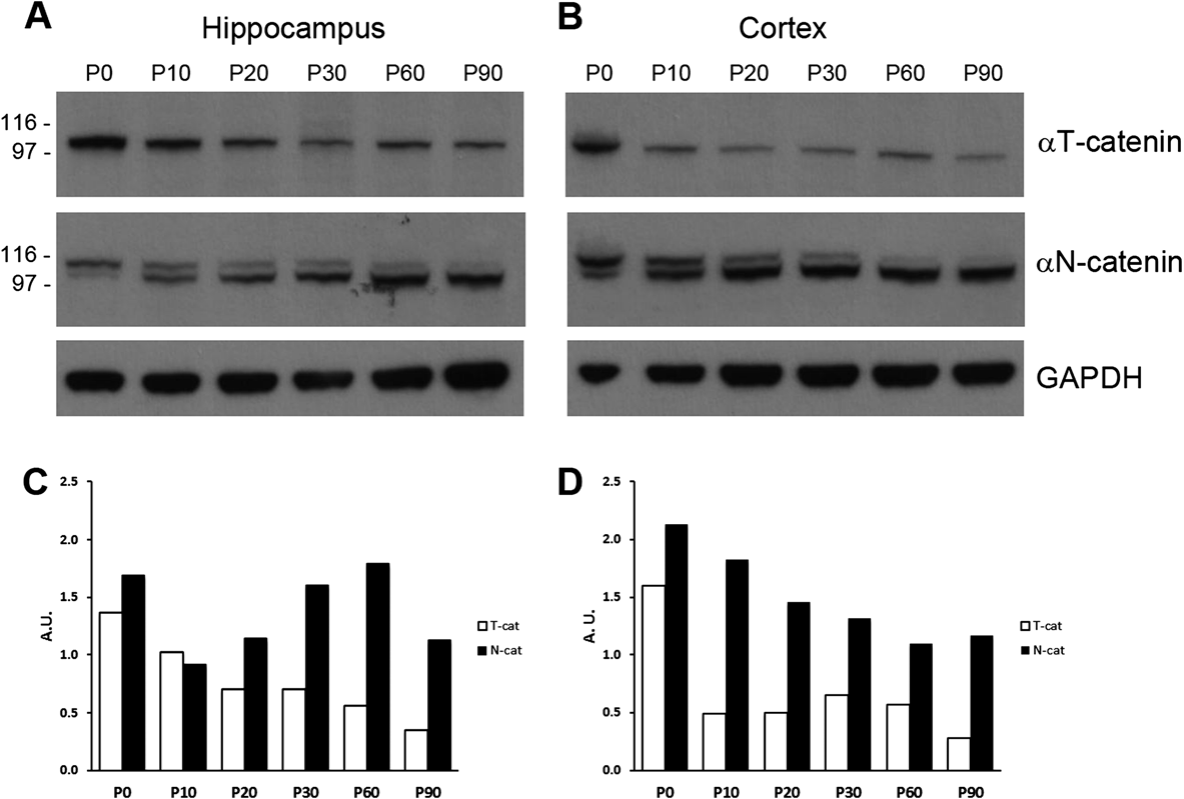
**Expression analysis of *ctnna3 *in mouse brain.** Western blot analysis of αT-catenin and αN-catenin in mouse hippocampus **(A)** and cortex **(B)** at different developmental stages (from P0 to P90). **(C**, **D)** Histograms showing the band intensity normalised by GAPDH as internal loading control.

Since the mouse *Ctnna3* cDNA encodes a protein showing 95% identity to human CTNNA3 and the genomic structures of the mouse *Ctnna3* and human *CTNNA3* genes are completely conserved, we have performed a Western blot analysis to get a quantitative characterisation of *Ctnna3* expression during early brain mouse development compared with the neural specific Ctnna2.Protein extracts of mouse cortex and hippocampus at different developmental stages (from P0 to P90) were probed with anti-N-catenin antibody that recognizes specifically the C-terminus of Ctnna2 as a doublet band and a rabbit polyclonal anti-αT-catenin antibody (#952), which recognizes a specific peptide corresponding to the C-terminus of Ctnna3. As shown in Figure 4, not only Ctnna2 is highly expressed at all brain developmental stages analysed, but also Ctnna3 is present in both brain areas analysed. Noticeably, Ctnna3 showed a higher expression in the hippocampus and cortex at P0, suggesting a specific neuronal role in very early developmental stages.

## Discussion

In a recent genome-wide scan performed by the AGP in the largest family-based sample of ASD reported to date [13], we have identified a compound heterozygous deletion encompassing the *CTNNA3* gene in a male proband with autism. This deletion removes a coding exon (exon11), leading to a putative frameshift and premature stop codon, and it is inherited from both parents, each heterozygote for a deletion of slightly different length. Given the likelihood of nonsense-mediated mRNA decay of *CTNNA3* transcripts carrying exon 11 deletions, this compound heterozygous deletion is thus expected to result in a complete lack of functional protein in the affected individual.

This discovery is of particular interest as *CTNNA3* is a very promising candidate gene for ASD based on its biological function, such as its crucial role in cell adhesion, a pathway previously implicated in ASD. *CTNNA3* encodes αT-catenin, a member of the α-catenin family of cell-cell adhesion molecules. Like other α-catenins, CTNNA3 provides an indispensable link between the cadherin-based cell-cell adhesion complex and the cytoskeleton to mediate cell-cell adhesion.

There are three alpha catenin genes: CTNNA1 (alpha E-catenin) is ubiquitously expressed but mainly in epithelial tissues [35], CTNNA2 (alpha N-catenin) shows a neural specific expression pattern [36], and CTNNA3 (alpha T-catenin) is expressed primarily in the heart and testis but at lower levels in the brain [30].

The role of CTNNA3 has been primarily investigated in the heart, due to its high expression in cardiac tissue and co-localization with plakophilin 2 [37]. Furthermore, genetic linkage and association studies have indicated that the *CTNNA3*/*LRRTM3* locus may play a role in susceptibility to late-onset Alzheimer's disease and plasma amyloid β levels [38-40]. However, the function of CTNNA3 in the brain remains largely unexplored.

Our Western blot characterisation of mouse Ctnna3 expression showed that it displays higher expression in the hippocampus and cortex at P0, suggesting a specific neuronal role in very early developmental stages. Thus, further studies are warranted in order to investigate the possible function of CTNNA3 in synapse adhesion [41].

It has been shown that Ctnna2 functions as a critical agent to regulate the stability and remodelling of synaptic contacts, as its loss causes deformation of dendritic spines, while its overexpression in dendrites causes an increase in spine and synapse density [42]. Overexpression of Ctnna3 and Ctnna1 also induced excess spine formation, suggesting that all α-catenin isoforms share the same spine-stabilizing activity [42]. Moreover, like other α-catenins, Ctnna3 participates in the canonical Wnt signalling pathway [43], which plays an important role in brain development and synaptic function. CNV and association studies investigating several genes involved in this pathway have provided evidence that Wnt signalling might be affected at least in a subset of individuals with ASD [44].

Beyond its biological plausibility, evidence for a potential role of *CTNNA3* in ASD susceptibility comes also from genetic studies. Two genome-wide association studies implicated common variants in *CTNNA3* in autism susceptibility [4,5]. Another study reported a *de novo* exonic deletion in *CTNNA3* associated to ASD, with additional evidence from transmission data [18]. Moreover, in a recent analysis of exon-disrupting CNVs affecting known autism candidate genes, *CTNNA3* was found borderline enriched in the autism cohort as opposed to controls (22 out of 2,588 autism cases versus 12 out of 2,670 controls, *P* = 0.050) [21]. Even if we have not confirmed this trend of enrichment for *CTNNA3* exonic deletions among children with ASD, no homozygous or compound heterozygous exonic deletions were found in a sample of 6,639 controls, suggesting that *CTNNA3* is haplosufficient and that only a recessive loss of function may play a role in ASD susceptibility. This hypothesis is consistent with the segregation of *CTNNA3* deletions in the discovery family 3456, as the unaffected sister inherited the exonic deletion in the heterozygous form. By sequence analysis of coding region of *CTNNA3* and the nested gene *LRRTM3* in four ASD families carrying exonic *CTNNA3* deletions, we could not confirm that these deletions act by unmasking rare variants in the non-deleted allele; however, we cannot exclude the possibility of point mutations in non-coding regions disrupting gene regulation or splicing.

Evidence of recessive inheritance in ASD comes from a study of consanguineous and multiplex ASD families [8,10-12] and from a recent case-control study [9], which estimated an overall 3% contribution to risk for ASD from recessive mutations. The proposed role of recessive mutations in ASD is also in accordance with the high heritability of ASD [45] and with the observation that the majority of parents of ASD individuals are both unaffected.

Notably, *CTNNA3*, one of the largest genes in the human genome, is located in a common fragile site (FRA10D) [32]. Increasing evidence links multiple fragile sites, which are considered hot spots for genomic instability, to neuropsychiatric diseases including autism [46,47].

Another feature that makes *CTNNA3* particularly interesting is the presence of a nested gene (*LRTMM3*), transcribed in the opposite direction. Interestingly, all three members of the alpha catenin family harbour leucine-rich repeat transmembrane neuronal (*LRRTM*) genes nested within the largest intron of each catenin family member. This structure is likely to derive from the insertion of an ancestral *lrrtm* gene into a pre-existing *ctnna* intron during early vertebrate evolution, followed by two subsequent duplications resulting in the three nested *lrrtm*/*ctnna* genes [48]. The LRRTM family members are brain-enriched transmembrane proteins, proposed to function as synaptic organizers during synapse development [49] by interacting with presynaptic neurexins [50]. *LRRTM3* displays expression predominantly in the brain, notably in the hippocampus, and, similar to *CTNNA3*, is thought to mediate cell adhesion [51,52]. The location of three *LRRTM* genes within α-catenin family members implies that transcriptional regulation of α-catenin genes and the respective *LRRTM*s could share common mechanisms. Therefore, it is plausible to hypothesize that deletions in *CTNNA3* could cause dysregulation of *LRRTM3* expression. However, given the CNS-restricted expression of *LRRTM3*, it was not possible to test this hypothesis in the patient's lymphocytes. Interestingly, a significant association of SNPs in *LRRTM3* with ASD in European populations was reported in a previous study [53], thus accumulating evidence that genetic variants in the *CTNNA3*/*LRRTM3* genomic region confer susceptibility to ASD.

Recently, gene targeting technology was used to delete the *Ctnna3* gene in the mouse to investigate the role of Ctnna3 in the heart where it is highly expressed. The *Ctnna3*-null mice are viable and fertile and show no obvious macroscopic phenotypic abnormality; however, they exhibit early-onset progressive dilated cardiomyopathy, gap junction remodelling and increased risk of cardiac arrhythmia [54]. To our knowledge, a detailed cognitive and behavioural characterisation of the *Ctnna3*-null mouse has not yet been carried out, which could provide important information about the presence of subtle impairments in behavioural assays relevant to the complex behaviours involved in autism [55]. In contrast to the mouse knock-out phenotype, when last evaluated, the child 3456_3 with the compound heterozygous *CTNNA3* deletion had a normal EKG and heart ultrasound. This suggests that CTNNA3 deficiency does not lead to a cardiac dysfunction in humans.

## Conclusions

Given the crucial role of catenins in both the development and maintenance of the nervous system, we believe that mutations affecting α-catenin's function may contribute to ASD pathogenesis. More specifically, our work implicates *CTNNA3* as a candidate gene in ASD, acting in a recessive mode of inheritance, and leads us to hypothesize that the identified compound heterozygous exonic deletion in *CTNNA3* causes ASD in family 3456. We also provide evidence that heterozygous exonic deletions in *CTNNA3* are not pathological. These results may be valuable in the context of clinical diagnosis and counselling.

## Competing interests

The authors declare that they have no competing interests.

## Authors’ contribution

EBa and EM designed the experimental plan, analysed the data and wrote the manuscript. FC and SL performed the mutation screening, allelic expression and CNV validation. DP provided and analysed CNV data in the control cohort. MG and PD carried out the expression analysis in the mouse cortex and hippocampus. EBo and PP provided the human brain tissues and participated in the allelic expression study. RT and AB provided the ASD discovery family and performed the clinical evaluation. All authors read and approved the final manuscript.

## Supplementary Material

Additional file 1: Figure S1 Junction fragments of maternal and paternal deletions in family 3456. DNA sequences, obtained from direct sequencing of the junction fragments, were aligned to the normal wild-type proximal and distal sequences. The presence of bases with perfect microhomology to the normal proximal and distal wild-type sequences is shown in *red*. Click here for file

Additional file 2: Table S1 *CTNNA3* exonic deletions in ASD cases and controls. Click here for file

Additional file 3: Figure S2 *CTNNA3* biallelic expression in the cerebellum and cerebral cortex. Sanger sequencing of rs4548513 (pSer596Asn) from genomic DNA (gDNA) and brain cDNA (cortex and cerebellum) of two adult controls showing the heterozygosity of the SNP and demonstrating biallelic expression of *CTNNA3* in both brain areas. Click here for file
