# Supplementary material for: A CTNNA3 compound heterozygous deletion implicates a role for αT-catenin in susceptibility to autism spectrum disorder
Source: J Neurodev Disord. 2014 Jul 10;6(1):17. doi: 10.1186/1866-1955-6-17 (PMC4104741; doi:10.1186/1866-1955-6-17)
Supplement: Additional file 1: Figure S1 — Junction fragments of maternal and paternal deletions in family 3456. DNA sequences, obtained from direct sequencing of the junction fragments, were aligned to the normal wild-type proximal and distal sequences. The presence of bases with perfect microhomology to the normal proximal and distal wild-type sequences is shown in red. [file 1866-1955-6-17-S1.pdf]

Alignment Maternal Deletion

```
Prox CCTATTTATTTTCTCACTGCTTTTCCTTCTGGAATTTCTAAAACAGGAAT
Del  CCTATTTATTTTCTCACTGCTTTTCCTTCTGGAATTTCTAAAACAGGAAT
Dist CAATGCTTTACCATCATCTGCCAAAGACGTGTGTGCAAATATCAGTCTTG

Prox AGACTATTTGTTTGCATAGTGATATCCCATATGTCATGTAAACTTTTTTC
Del  AGACTATTTGTTTGCATAGTGATATTGAGGCTAACCTGCCATCTGCTTAT
Dist CAAACAGACAATATTTGCACTACATTGAGGCTAACCTGCCATCTGCTTAT

Prox ATTTTTATTCTTTTTTCTCTTTTTGCCTAATTCGCTTATTTCAAAAGATC
Del  CAGATGTTCGGAGTCCAAATTGTACAGTTTTCCTTGTGTTTCCCCTAATG
Dist CAGATGTTCGGAGTCCAAATTGTACAGTTTTCCTTGTGTTTCCCCTAATG
```

Alignment Paternal Deletion

```
Prox GTGTAGGAACAGTACATACAACAAGGCATCTTTTAAAGGACAGAGTATTT
Del  GTGTAGGAACAGTACATACAACAAGGCATCTTTTAAAGGACAGAGTATTT
Dist TATACTTCACATTTACAGTAATTACTATGGCTTTACTTTCATTTGCTTCT

Prox CCTGAACTACATATTAATGTTATTTAGACAGATTTTTTAAAGTTTGATA
Del  CCTGAACTACATATTAATGTTATTTAATATTAAATGTTATTTAATATTTA
Dist CTCAGTTTTCCTTCATCTTGCAACAAGACA--------------------

Prox TGTACATCAATATAAAATTTAAAAGGGAGTCATGGGTGGGGGAAATGGGA
Del  ATATCAAGAAAAAGCAGGTAGTAGGTAGCTTCTAACAGAAGAACAGACAA
Dist --ATCAAGAAAAAGCAGGTAGTAGGTAGCTTCTAACAGAAGAACAGACAA

Prox AAATGCTGGTCAAGGTGTACAAAGTTTCATTTAGGTAGCA
Del  AAGAGAGCATAAAGAAGATATTAGCCAAGAAGTGATGCTT
Dist AAGAGAGCATAAAGAAGATATTAGCCAAGAAGTGATGCTT
```
