# Supplementary material for: A CTNNA3 compound heterozygous deletion implicates a role for αT-catenin in susceptibility to autism spectrum disorder
Source: J Neurodev Disord. 2014 Jul 10;6(1):17. doi: 10.1186/1866-1955-6-17 (PMC4104741; doi:10.1186/1866-1955-6-17)
Supplement: Additional file 2: Table S1 — CTNNA3 exonic deletions in ASD cases and controls. [file 1866-1955-6-17-S2.docx]

**Supplementary Table S1**

|  | |  | |  | |  | |  | |  | |  |
| --- | --- | --- | --- | --- | --- | --- | --- | --- | --- | --- | --- | --- |
| **a) *CTNNA3* exonic deletions in ASD cases** | | | | | |  | |  | |  | |  |
| **Sample ID** | **Inheritance** | | **Array_platform** | | **Genomic coordinates (hg18)^a^** | | **Length (bp)** | | **Deleted exons^b^** | | **Effect on CDS** | |
| 5237_3 | paternal | | illumina 1Mv1 single | | chr10:67628183-67752490 | | 124308 | | x13 | | frameshift | |
| 3311_003 | maternal | | illumina 1Mv1 single | | chr10:67688367-67759307 | | 70941 | | x13 | | frameshift | |
| **3456_3** | **maternal/paternal** | | **Illumina 1Mv3 duo** | | **chr10:67898172-68002674^c^** | | **104502** | | **x11** | | **frameshift** | |
| 4196_1 | mother | | illumina 1Mv1 single | | chr10:67912678-67976820 | | 64143 | | x11 | | frameshift | |
| 4527_1 | mother | | illumina 1Mv1 single | | chr10:67947835-67968052 | | 20218 | | x11 | | frameshift | |
| 4228_1 | N/A | | illumina 1Mv1 single | | chr10:67957765-68226404 | | 268640 | | x10 | | in frame | |
| 6372_3 | maternal (father N/A) | | Illumina 1Mv3 duo | | chr10:67979174-68114740 | | 135567 | | x10 | | in frame | |
| 14299_4200 | paternal | | illumina 1Mv1 single | | chr10:67987089-68056520 | | 69432 | | x10 | | in frame | |
| 3093_004 | maternal | | illumina 1Mv1 single | | chr10:67987089-68067310 | | 80222 | | x10 | | in frame | |
| 5013_3 | paternal | | illumina 1Mv1 single | | chr10:68023745-68071037 | | 47293 | | x10 | | in frame | |
| 3169_004 | maternal | | illumina 1Mv1 single | | chr10:68029140-68183933 | | 154794 | | x10 | | in frame | |
| 4211_1 | father | | illumina 1Mv1 single | | chr10:68029140-68183933 | | 154794 | | x10 | | in frame | |
| 4291_1 | N/A | | illumina 1Mv1 single | | chr10:68052141-68210655 | | 158515 | | x8-x9 | | in frame | |
| 5065_3 | maternal | | illumina 1Mv1 single | | chr10:68138586-68227559 | | 88974 | | x8-x9 | | in frame | |
| 3476_3 | paternal | | Illumina 1Mv3 duo | | chr10:68154851-68247375 | | 92525 | | x8-x9 | | in frame | |

| **b) *CTNNA3* exonic deletions in controls** | | |  |  |  |  |
| --- | --- | --- | --- | --- | --- | --- |
| **Sample ID** | **Control_cohort** | **Array_platform** | **Genomic coordinates (hg18)** | **Length (bp)** | **Deleted exons** | **Effect on CDS** |
| B601040_1007870358 | SAGE | illumina 1Mv1 single | chr10:67628183-67752490 | 124308 | x13 | frameshift |
| B782997_1007853703 | SAGE | illumina 1Mv1 single | chr10:67628183-67754983 | 126801 | x13 | frameshift |
| B974175_1007875270 | SAGE | illumina 1Mv1 single | chr10:67748487-67889985 | 141499 | x12 | in frame |
| CONSPC2_f_179516 | POPGEN | Affy6 | chr10:67754797-67871675 | 116879 | x12 | in frame |
| B355026_0067942568 | SAGE | illumina 1Mv1 single | chr10:67820257-68046869 | 226613 | x11 | frameshift |
| B291548_1007841762 | SAGE | illumina 1Mv1 single | chr10:67834450-68127819 | 293370 | x11 & x10 | frameshift |
| CONSPC_m_183371 | POPGEN | Affy6 | chr10:67911535-67975943 | 64409 | x11 | frameshift |
| HABC_900384_900384 | HABC | Illumina 1Mv3 duo | chr10:67912678-67968052 | 55375 | x11 | frameshift |
| HABC_900268_900268 | HABC | Illumina 1Mv3 duo | chr10:67920659-68059454 | 138796 | x11 & x10 | frameshift |
| CONT1635 | OHI | Affy6 | chr10:67937332-67971629 | 34298 | x11 | frameshift |
| CONT-1870 | OHI | Affy6 | chr10:67938271-68044090 | 105820 | x11 | frameshift |
| 110036018737 | OC | illumina 1Mv1 single | chr10:67938287-68059454 | 121168 | x11 & x10 | frameshift |
| Caucasian | CHOP | Illumina 550K | chr10:67944050-68069165 | 125116 | x10-x11 | frameshift |
| Caucasian | CHOP | Illumina 550K | chr10:67944050-68077867 | 133818 | x10-x11 | frameshift |
| Caucasian | CHOP | Illumina 550K | chr10:67944050-68088852 | 144803 | x10-x11 | frameshift |
| CONT1792 | OHI | Affy6 | chr10:67955244-68119974 | 164731 | x10 | in frame |
| Caucasian | CHOP | Illumina 550K | chr10:67960600-68052141 | 91542 | x10 | in frame |
| CONT2294 | OHI | Affy6 | chr10:67981580-68139445 | 157866 | x10 | in frame |
| B431282_1007873513 | SAGE | illumina 1Mv1 single | chr10:67987089-68088852 | 101764 | x10 | in frame |
| Caucasian | CHOP | Illumina 550K | chr10:67997021-68053979 | 56959 | x10 | in frame |
| HABC_900966_900966 | HABC | Illumina 1Mv3 duo | chr10:67997021-68091312 | 94292 | x10 | in frame |
| CONT-2085 | OHI | Affy6 | chr10:68009168-68066669 | 57502 | x10 | in frame |
| HABC_901209_901209 | HABC | Illumina 1Mv3 duo | chr10:68013385-68156906 | 143522 | x10 | in frame |

| **Sample ID** | **Control_cohort** | **Array_platform** | **Genomic coordinates (hg18)** | **Length (bp)** | **Deleted exons** | **Effect on CDS** |
| --- | --- | --- | --- | --- | --- | --- |
| CONSPC_f_186937 | POPGEN | Affy6 | chr10:68020146-68070424 | 50279 | x10 | in frame |
| B779950_1007875276 | SAGE | illumina 1Mv1 single | chr10:68023745-68071037 | 47293 | x10 | in frame |
| B941932_1007873623 | SAGE | illumina 1Mv1 single | chr10:68023745-68071037 | 47293 | x10 | in frame |
| HABC_900941_900941 | HABC | Illumina 1Mv3 duo | chr10:68023745-68127819 | 104075 | x10 | in frame |
| NCA07346 | OHI | Affy6 | chr10:68024710-68070424 | 45715 | x10 | in frame |
| CONT1593 | OHI | Affy6 | chr10:68024710-68075715 | 51006 | x10 | in frame |
| Caucasian | CHOP | Illumina 550K | chr10:68034046-68088852 | 54807 | x10 | in frame |
| CONBSP_m_213905 | POPGEN | Affy6 | chr10:68037680-68109008 | 71329 | x10 | in frame |
| Caucasian | CHOP | Illumina 550K | chr10:68040186-68088852 | 48667 | x10 | in frame |
| CONSPC2_m_195755 | POPGEN | Affy6 | chr10:68050416-68077020 | 26605 | x10 | in frame |
| B764705_1007853643 | SAGE | illumina 1Mv1 single | chr10:68091312-68222395 | 131084 | x9 & x8 | in frame |
| HABC_902495_902495 | HABC | Illumina 1Mv3 duo | chr10:68113705-68200470 | 86766 | x9 | in frame |
| Caucasian | CHOP | Illumina 550K | chr10:68127614-68216254 | 88641 | x8-x9 | in frame |
| Caucasian | CHOP | Illumina 550K | chr10:68160982-68305039 | 144058 | x8-x9 | in frame |
| Caucasian | CHOP | Illumina 550K | chr10:68180377-68268934 | 88558 | x8-x9 | in frame |
| Caucasian | CHOP | Illumina 550K | chr10:68190451-68252582 | 62132 | x8-x9 | in frame |
| NCA07559 | OHI | Affy6 | chr10:68197491-68520899 | 323409 | x8^d^ | in frame |
| CONT-1970 | OHI | Affy6 | chr10:68211267-68626505 | 415239 | x7^e^ | frameshift |
| CONBSP_f_186933 | POPGEN | Affy6 | chr10:68622864-68770434 | 147571 | x6 | in frame |

| ^a^ The size of CNV shown is as detected by microarrays |  |
| --- | --- |
| ^b^ Exons numbering according to RefSeq NM_013266  ^c^ Exact breakpoint coordinates |  |
| ^d^ The deletion includes also exon1 of LRRTM3 |  |
| ^e^ The deletion includes also exon1 and exon2 of LRRTM3 | |
