# Supplementary figures and images for: A CTNNA3 compound heterozygous deletion implicates a role for αT-catenin in susceptibility to autism spectrum disorder
Source: J Neurodev Disord. 2014 Jul 10;6(1):17. doi: 10.1186/1866-1955-6-17 (PMC4104741; doi:10.1186/1866-1955-6-17)

Control 1

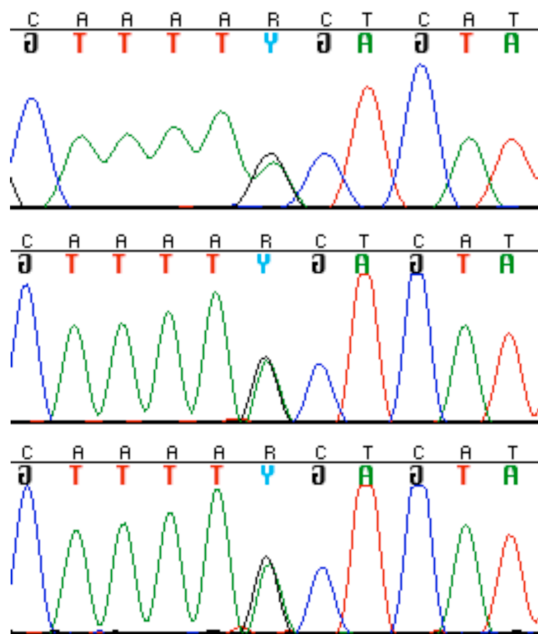

Control 2

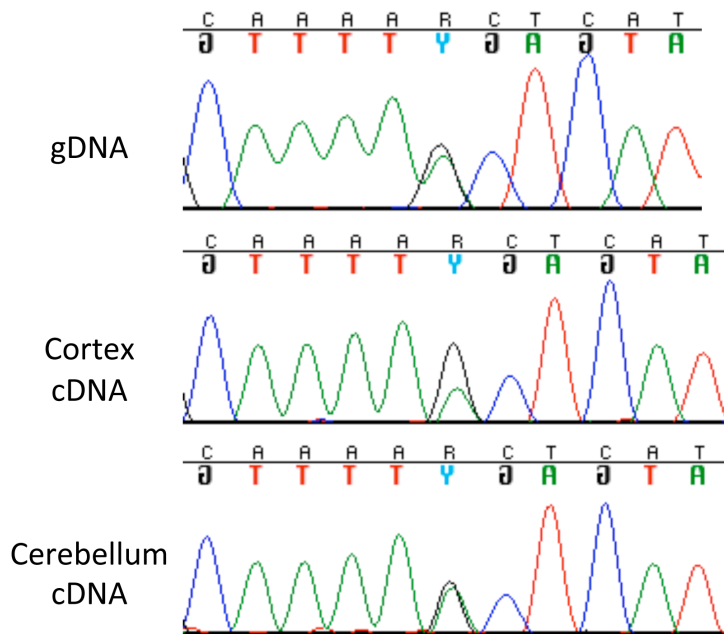

Supplement: Additional file 3: Figure S2 — CTNNA3 biallelic expression in the cerebellum and cerebral cortex. Sanger sequencing of rs4548513 (pSer596Asn) from genomic DNA (gDNA) and brain cDNA (cortex and cerebellum) of two adult controls showing the heterozygosity of the SNP and demonstrating biallelic expression of CTNNA3 in both brain areas. [file 1866-1955-6-17-S3.pdf]
